# Retinal Functional and Structural Neural Indices: Potential Biomarkers for the Monitoring of Cerebral Neurodegeneration: The Maastricht Study

**DOI:** 10.3233/JAD-230104

**Published:** 2023-06-13

**Authors:** Frank C.T. van der Heide, Sara Mokhtar, Anjani Khanna, Mozhda Said, Ronald M.A. Henry, Abraham A. Kroon, Pieter C. Dagnelie, Simone J.P.M. Eussen, Tos T.J.M. Berendschot, Jan S.A.G. Schouten, Miranda T. Schram, Carla J.H. van der Kallen, Marleen M.J. van Greevenbroek, Anke Wesselius, Hans H.C.M. Savelberg, Nicolaas C. Schaper, Carroll A.B. Webers, Coen D.A. Stehouwer

**Affiliations:** a CARIM School for Cardiovascular Diseases, Maastricht University (UM), The Netherlands; b Department of Internal Medicine, Maastricht University Medical Centre+ (MUMC+), The Netherlands; c MHeNS School of Mental Health and Neuroscience, Maastricht University (UM), The Netherlands; d University Eye Clinic Maastricht, MUMC+, TheNetherlands; e Department of Ophthalmology, Sharpsight eye hospital, New Delhi, India; f Heart and Vascular Centre, MUMC+ Maastricht, The Netherlands; g Department of Epidemiology, UM, The Netherlands; h CAPHRI Care and Public Health Research Institute, UM, The Netherlands; i Department of Ophthalmology, Canisius Wilhelmina Hospital, Nijmegen, The Netherlands; j Department of Genetics and Cell Biology, Complex Genetics, UM, The Netherlands; k NUTRIM School for Nutrition and Translational Research in Metabolism, UM, The Netherlands; l Department of Nutrition and Movement Sciences, UM, The Netherlands; m Department of Social Medicine, MUMC+, The Netherlands; n Department of Internal Medicine, Division of Endocrinology and Metabolic Disease, Maastricht University Medical Centre+, Maastricht, The Netherlands

**Keywords:** Alcohol consumption, Alzheimer’s disease, cardiorespiratory fitness, dementia, imaging biomarkers, obesity, optical coherence tomography, perimetry, physical inactivity, retinal neurodegeneration

## Abstract

**Background:**

If retinal indices of neurodegeneration are to be biomarkers for the monitoring of cerebral neurodegeneration, it is important to establish whether potentially modifiable risk factors for dementia are associated with retinal neurodegenerative changes.

**Objective:**

To study associations of dementia risk factors with retinal sensitivity, an index of retinal neural function, and retinal nerve fiber layer (RNFL) thickness, an index of retinal neural structure.

**Methods:**

We used cross-sectional data from The Maastricht Study (up to 5,666 participants, 50.5% men, mean age 59.7), and investigated associations with regression analyses (adjusted for potential confounders).

**Results:**

Most risk factors under study (i.e., hyperglycemia, unhealthy diet, lower cardiorespiratory fitness, smoking, alcohol consumption, and hypertension) were significantly associated with lower retinal sensitivity and lower RNFL thickness.

**Conclusion:**

Findings of this population-based study support the concept that retinal neural indices may be biomarkers for the monitoring of therapeutic strategies that aim to prevent early-stage cerebral neurodegeneration and, ultimately, dementia.

## INTRODUCTION

Clinical dementia, the end stage of cerebral neurodegeneration, is preceded by a gradual loss of neurons over time [1–3]. Mechanistically, deterioration of microvascular and neuronal structures is thought to hamper the function of the neurovascular coupling unit, which can impair autoregulation, and subsequently result in neural ischemia and neural exposure to toxins, both of which can induce neurodegeneration [1, 2]. As postulated in the ticking clock hypothesis [4, 5], there may be an opportunity to prevent the onset and/or progression of early microvascular and neuronal deterioration via reducing early exposure to potentially modifiable risk factors for these detrimental changes [3–5]. Such risk factors include hyperglycemia, an unhealthy diet, lower cardiorespiratory fitness, excessive alcohol consumption, smoking, hypertension, dyslipidemia, obesity, and lower levels of physical activity[6–13].

Currently, there are no clinical tools available to, at an individual level, monitor the efficacy of therapeutic strategies that aim to prevent early-stage cerebral neurodegeneration in the absence of clinical dementia [14, 15]. This is an important issue because early monitoring may facilitate personalized, targeted prevention of early-stage cerebral neurodegeneration [14, 15].

The retina may provide an opportunity to monitor therapeutic strategies that aim to prevent early-stage neurodegenerative changes [16]. Retinal measures of neuronal function and structure are biologically plausible biomarkers for the monitoring of cerebral neurodegenerative changes because the retina and the brain have a shared embryology, and both have many anatomical and physiological similarities [16]. Indeed, early structural retinal neurodegenerative changes have been found to be associated with MRI-assessed markers of cerebral neurodegeneration (i.e., lower grey and white matter volume) [17], cognitive decline [18], and dementia [19].

In the retina, early functional and structural neurodegenerative changes can, respectively, be non-invasively and accurately assessed as lower retinal sensitivity and lower retinal nerve fiber layer (RNFL) thickness [16, 20]. Loss of retinal sensitivity reflects greater dysfunction of the neural networks which perceive, filter, and transmit visual information from the retina to the brain [20]. RNFL thinning reflects loss of retinal ganglion cell axons, which transmit visual information from the retina to the brain [16].

Current literature on whether potentially modifiable risk factors for dementia may be determinants of retinal sensitivity or RNFL thickness has important limitations [21–32]. First, no large population-based studies have investigated the associations of potentially modifiable risk factors for dementia with retinal sensitivity. Second, previous studies have not yet investigated the associations with RNFL thickness of a number of important potentially modifiable risk factors for dementia, i.e., adherence to a healthy diet, cardiorespiratory fitness, and accelerometer-assessed lower physical activity [22–32].

In view of above, we investigated, using a large, well-characterized population-based cohort study, whether potentially modifiable risk factors for dementia are associated with retinal sensitivity and RNFL thickness.

## MATERIALS AND METHODS

Here key elements of the Material and Methods are provided, more details are provided in the Supplementary Methods.

### Study population and design

We used data from The Maastricht Study, a population-based observational cohort study. The rationale and methodology have been described previously [33]. In brief, the study focuses on the etiology, pathophysiology, complications, and comorbidities of type 2 diabetes mellitus and is characterized by an extensive phenotyping approach. Eligible for participation were all individuals aged between 40 and 75 years and living in the southern part of the Netherlands. Recruitment was stratified according to known type 2 diabetes status, with an oversampling of individuals with type 2 diabetes, for reasons of efficiency [33]. The present report includes cross-sectional data of 7,689 participants who completed the baseline survey between November 2010 and December 2017.

### Retinal sensitivity

We assessed retinal sensitivity of both eyes in the central and peri macular area with the Heidelberg Edge Perimeter (Heidelberg Engineering, Heidelberg, Germany). In brief, light stimuli varying in strength between 0 and 35 decibels were presented at 54 coordinates on the retina; at each coordinate the threshold of visual perception (i.e., the threshold at which the weakest presented visual stimulus could be perceived) was determined; and results were averaged into ‘retinal sensitivity’. The intra-observer reliability for the assessment of the retinal sensitivity is 0.95 [34].

### RNFL thickness

We assessed RNFL thickness with optical coherence tomography (OCT; Spectralis unit; Heidelberg Engineering, Heidelberg, Germany). The RNFL thickness (μm) of both eyes was measured within a 3.45 mm diameter circular scan (12°, 768 voxels, 100 automatic real-time tracking) centered on the optic nerve head. All OCT scans were reviewed, and their quality was scored. Intra- and interindividual reliability, expressed as intraclass correlation coefficients, were 0.97 and 0.96, respectively [35].

### Assessment of risk factors

We determined hemoglobin A1c (HbA1c; % or mmol/mol) and total cholesterol (mmol/L) in fasting venous plasma samples [33]; assessed dietary intake, including alcohol consumption, with a validated food frequency questionnaire [36], and calculated the Dutch Healthy Diet index sum score (without alcohol consumption) [36, 37]; and categorized alcohol consumption into none, light, moderate, and high (definitions in Supplementary Material) [38]. Then, we assessed cardiorespiratory fitness, defined as the maximum power output adjusted for body mass (i.e., *W*_max_·kg^–1^), with a graded cycle ergometer-, submaximal exercise test [39]; assessed smoking status (current, former, never smoking) via a questionnaire [33]; assessed antihypertensive medication use, an index of past exposure to a relatively high blood pressure, as part of an interview, assessed 24-h ambulatory blood pressure (mm Hg) with an oscillometric device [40]; calculated mean arterial pressure from the 24-h ambulatory blood pressure measurements as ([1/3*systolic 24-h ambulatory blood pressure] + [2/3*diastolic 24-h ambulatory blood pressure]); assessed waist circumference (cm) as part of a physical examination [33]; and measured 8-day physical activity (h/day) with an accelerometer [41].

### Covariates

As described previously [33], we assessed educational level (low, intermediate, high) by questionnaire [42], high-density lipoprotein (HDL) and fasting plasma glucose in fasting venous blood samples [33]; assessed medication use as part of an interview, and assessed glucose metabolism status based on fasting plasma glucose and oral glucose tolerance test-derived 2-h post load glucose [33].

### Statistical analyses

We used multivariable regression analysis to investigate the associations of potentially modifiable risk factors for dementia (determinants) with retinal sensitivity and RNFL thickness (outcomes). We standardized determinants and outcomes of a continuous nature (i.e., expressed as z-score) and entered categorical variables into models as dummy variables. Next, we inversed (i.e., multiplied by –1) the healthy diet score, cardiorespiratory fitness, and physical activity so that higher values indicate lower healthy diet score, lower cardiorespiratory fitness, or lower physical activity. Last, we used complete case analysis, where individuals were included in the main analyses if data were available on the main determinant, the outcome, and potential confounders required for the fully adjusted model(model 3).

We adjusted for potential confounders. In model 1 we did not adjust for any confounders (“crude”). In model 2, we adjusted for demographic confounders (i.e., age, sex, and educational status) and for glucose metabolism status [32]. In model 3, we additionally adjusted for cardiovascular and lifestyle variables (i.e., office systolic blood pressure, antihypertensive medication use [yes/no], waist circumference, total cholesterol/HDL cholesterol ratio, lipid-lowering medication use [yes/no], smoking status [current, former, never], and alcohol consumption status [none, moderate, high]) [32]. For associations where HbA1c was the determinant, glucose metabolism status was not entered into the model to prevent collinearity. The associations were expressed as standardized regression coefficient (stβ) and corresponding 95% confidence interval(95% CI).

We tested for interaction by sex and glucose metabolism status to assess whether associations, respectively, differed between men and women or between individuals with type 2 diabetes, prediabetes, and normal glucose metabolism. For interaction analyses with glucose metabolism status, we excluded participants with other types of diabetes because the number of these participants was small.

To assess the robustness of our findings we performed a range of additional analyses (we report one additional analysis in the main manuscript; and report all additional analyses in the Supplementary Methods). We studied the associations of age with retinal sensitivity and RNFL thickness. We performed these analyses so that we could compare with how many years of “aging” the betas for determinants under study correspond.

All analyses were performed with Statistical Package for Social Sciences version 25.0 (IBM SPSS, IBM Corp, Armonk, NY, USA). For all analyses, a *p*-value <0.05 was considered statisticallysignificant.

## RESULTS

### Selection and characteristics of the study population

**Fig. 1:**
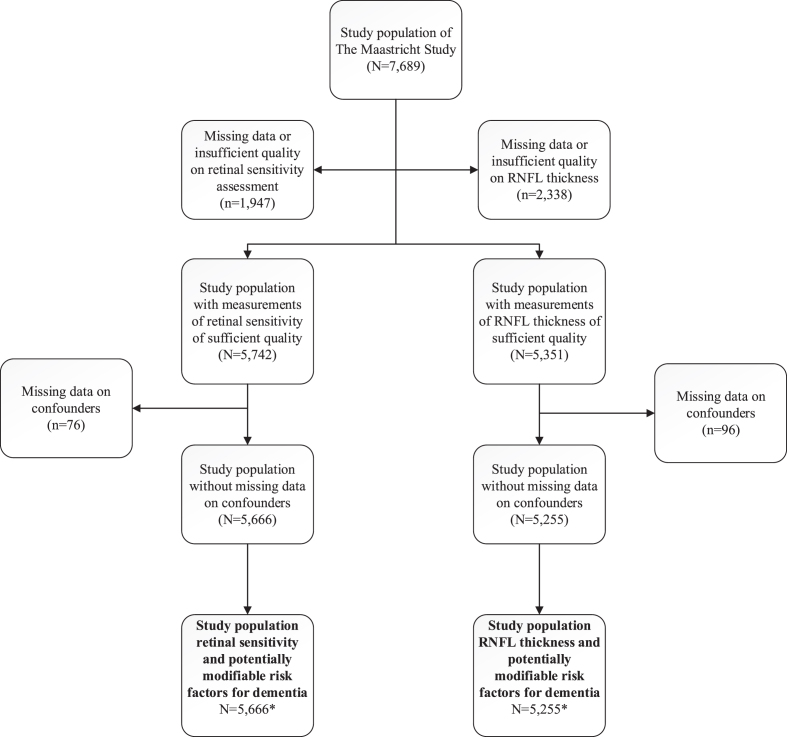
delineates the selection of participants for inclusion in analyses. *For retinal sensitivity and RNFL thickness, respectively, the numbers of participants with complete data for analyses with HbA1c were *n* = 5,662 and *n* = 5,249; for analyses with the healthy diet score were *n* = 5,369 and *n* = 4,981; for analyses with cardiorespiratory fitness were *n* = 4,899 and *n* = 4,452; for analyses with alcohol consumption were *n* = 5,377 and *n* = 4,989; for analyses with smoking were *n* = 5,666 and *n* = 5,255; for analyses with antihypertensive medication use were *n* = 5,666 and *n* = 5,255; for analyses with 24-h ambulatory blood pressure were *n* = 5,074 and *n* = 4,746; for analyses with total cholesterol were *n* = 5,664 and *n* = 5,255; for analyses with waist circumference were *n* = 5,666 and *n* = 5,255; and for analyses with physical activity were *n* = 5,027 and *n* = 4,510. Confounders are: age, sex, glucose metabolism status, educational level, office systolic blood pressure (where applicable), antihypertensive medication use, waist circumference (where applicable), total cholesterol/ HDL ratio (where applicable), lipid-lowering medication use, smoking (where applicable), and alcohol consumption (where applicable; a precise overview of covariates entered per model [for all analyses] is presented in the Methods). RNFL, retinal nerve fiber layer; HbA1c, glycated hemoglobin.

Figure 1 shows an overview of the study population selection.

Table 1 and Supplementary Tables 1 and 2 show general participant characteristics according to tertiles of retinal sensitivity and RNFL thickness. Overall, participants with a lower retinal sensitivity and thinner RNFL were older, and generally had a more adverse risk factor profile. General characteristics of participants included in the study were comparable to those of participants with missing data (Supplementary Table 3).

**Table 1:** General study population characteristics according to tertiles of retinal sensitivity in the study population with complete data on waist circumference

Characteristic	Retinal sensitivity			
	Total study group	Tertile 1 (low)	Tertile2 (middle)	Tertile 3 (high)
	(N = 5,666)	(N = 1,877)	(N = 1,901)	(N = 1,888)
Demographic characteristics				
Age (y)	59.7±8.7	64.2±7.9	59.6±8.2	55.4±7.8
Men	2862 (50.5)	936 (49.%)	960 (50.5)	966 (51.2)
Educational level				
Low	1927 (34.0)	809 (43.1)	618 (32.5)	500 (26.5)
Middle	1571 (27.7)	463 (24.7)	525 (27.6)	583 (30.9)
High	2168 (38.3)	605 (32.2)	758 (39.9)	805 (42.6)
Potentially modifiable risk factors for dementia				
HbA1c (mmol/mol)*	39.1±9.3	40.9±9.8	38.7±9.1	37.9±8.8
HbA1c (%)*	5.7±0.9	5.9±0.9	5.7±0.8	5.6±0.8
Dutch Healthy Diet score (points)*	76.6±14.6	77.1±14.6	76.9±14.6	75.8±14.5
Cardiorespiratory fitness (Wmax·kg– 1)	2.1±0.6	2.0±0.6	2.2±0.6	2.3±0.6
Alcohol consumption				
None	814 (15.1)	305 (17.1)	240 (13.2)	269 (15.2)
Light	1656 (17.6)	533 (29.8)	548 (30.2)	574 (32.4)
Moderate	1186 (22.1)	358 (20.0)	396 (21.8)	430 (24.3)
High	1721 (32.0)	592 (33.1)	631 (34.8)	497 (28.1)
Smoking status				
Never	2170 (38.3)	662 (35.3)	732 (38.5)	776 (41.1)
Former	2785 (49.2)	981 (52.3)	934 (49.1)	870 (46.1)
Current	711 (12.5)	234 (12.5)	235 (12.4)	242 (12.8)
Antihypertensive medication use	2074 (36.6)	895 (47.7)	665 (35.0)	514 (27.0)
Ambulatory 24-h systolic blood pressure (mmHg)*	118.9±11.6	120.1±12.1	118.6±11.2	117.7±11.3
Ambulatory 24-h diastolic blood pressure (mmHg)*	72.9±7.2	72.0±7.1	73.1±7.1	73.3±7.3
Mean arterial pressure (mm Hg)	88.2±8.0	88.1±8.0	88.2±7.8	88.4±8.1
Total cholesterol (mmol/L)*	5.2±1.1	5.1±1.1	5.2±1.1	5.3±1.1
Waist circumference (cm)	94.8±13.4	96.3±13.5	94.4±13.2	93.5±13.3
Physical activity (minutes per day)*	118.9±40.9	115.0±39.7	121.4±41.3	120.3±41.2
Other				
Glucose metabolism status				
Normal glucose metabolism	3514 (62.0)	995 (53.0)	1199 (63.1)	1320 (69.9)
Prediabetes	840 (14.8)	319 (17.0)	292(15.4)	229 (12.1)
Type 2 diabetes	1278 (22.6)	552 (29.4)	400 (21.0)	326 (17.3)
Other type of diabetes	34 (0.6)	11 (0.6)	10 (0.5)	13 (0.7)
Glucose-lowering medication use	957 (16.9)	414 (21.1)	299 (15.7)	244 (12.9)
Lipid-lowering medication use	1744 (30.8)	781 (41.6)	550 (28.9)	413 (21.9)
Total/HDL cholesterol ratio (no unit)	3.6±1.2	3.5±1.1	3.6±1.2	3.6±1.2
Outcomes				
Retinal sensitivity (dB)	27.7±1.6	26.1±1.6	27.9±0.3	29.1±0.5
RNFL thickness (μm)*	94.9±10.8	83.3±6.8	95.3±2.5	106.1±6.3

Data are presented as mean±standard deviation, median [interquartile range] or number (%). ^*^Data shown in the study population with complete data on ambulatory 24-h blood pressure (*n* = 5,074), total cholesterol (*n* = 5,664), HbA1c (*n* = 5,662), Dutch Healthy Diet score (*n* = 5,369), alcohol consumption (*n* = 5,377 [shown for alcohol consumption assessed with the food frequency questionnaire]), cardiorespiratory fitness (*n* = 4,899), physical activity (*n* = 5,027), and RNFL thickness (*n* = 5,255). HDL, high-density lipoprotein; HbA1c, glycated hemoglobin A1c.

### Associations of risk factors with retinal sensitivity and RNFL thickness

#### HbA1c

**Fig. 2:**
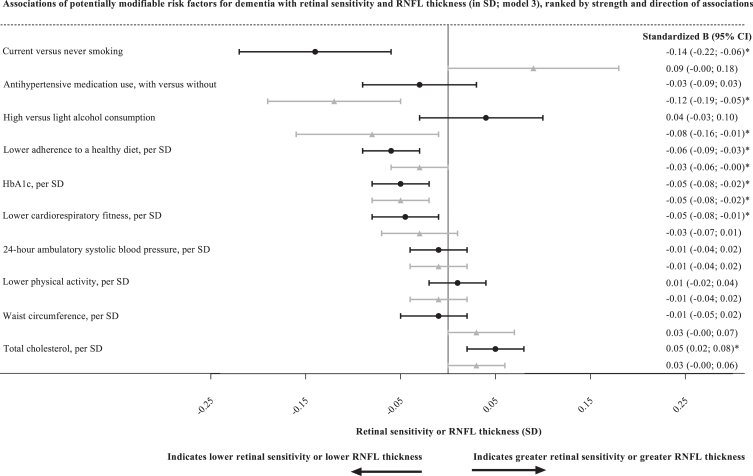
Associations of potentially modifiable risk factors for dementia with retinal sensitivity and RNFL thickness, ranked by strength and direction of associations. Standardized regression coefficients (stβ) represent the difference in retinal sensitivity (black circular point estimates with error bars) or RNFL thickness (grey triangular point estimated with error bars) in SD for one SD greater HbA1c, lower healthy diet score, lower cardiorespiratory fitness, for high versus light total alcohol consumption, for current versus never smoking, for with versus without antihypertensive medication use, greater 24-h ambulatory systolic blood pressure, greater total cholesterol, greater waist circumference, or lower physical activity. Numerical values with which 1 SD corresponds are presented in the legend of Table 2. *denotes *p* < 0.05. Variables in models: age, sex, glucose metabolism status (where applicable), educational level, office systolic blood pressure (where applicable), antihypertensive medication use (where applicable), waist circumference (where applicable), total cholesterol/ HDL ratio (where applicable), lipid-lowering medication use, smoking (where applicable), and alcohol consumption (where applicable; a precise overview of covariates entered per model [for all analyses] is presented in the Methods). RNFL, retinal nerve fiber layer; SD, standard deviation; CI, confidence interval; HbA1c, hemoglobin A1c; HDL, high density lipoprotein.

After full adjustment (model 3), greater HbA1c was significantly associated with lower retinal sensitivity and lower RNFL thickness (per SD, standardized beta [95% CI], –0.05 [–0.08; –0.02], and –0.05 [–0.08; –0.02], respectively; Table 2 and Fig. 2).

**Table 2:** Associations of potentially modifiable risk factors for dementia with retinal sensitivity and RNFL thickness

		Retinal Sensitivity, per SD		RNFL thickness, per SD	
	Model	Number of participants in analyses	stβ (95% CI)	Number of participants in analyses	stβ (95% CI)
Potentially modifiable risk factors for dementia				
HbA1c, per SD	1	N = 5,662	**–0.15 (–0.17; –0.12)**	N = 5,249	**–0.06 (–0.09; –0.04)**
	2		**–0.07 (–0.10; –0.05)**		**–0.05 (–0.08; –0.02)**
	3		**–0.05 (–0.08; –0.02)**		**–0.05 (–0.08; –0.02)**
Lower healthy diet score, per SD	1	N = 5,369	–0.01 (–0.03; 0.02)	N = 4,981	**–0.04 (–0.07; –0.01)**
	2		**–0.07 (–0.09; –0.04)**		–0.03 (–0.06; 0.00)	
	3		**–0.06 (–0.09; –0.03)**		**–0.03 (–0.06; –0.00)**
Lower cardiorespiratory fitness, per SD	1	N = 4,899	**–0.21 (–0.23; –0.18)**	N = 4,542	–0.01 (–0.04; 0.02)
	2		**–0.05 (–0.08; –0.02)**		–0.02 (–0.05; 0.02)
	3		**–0.05 (–0.08; –0.01)**		–0.03 (–0.07; 0.01)
Alcohol consumption		N = 5,377		N = 4,989	
- None versus light	1		**–0.10 (–0.19; –0.02)**		0.05 (–0.04; 0.13)
	2		–0.05 (–0.13; 0.03)		0.01 (–0.08; 0.10)
	3		–0.03 (–0.11; 0.05)		0.01 (–0.08; 0.09)
- Moderate versus light	1		0.07 (–0.01; 0.14)		0.03 (–0.05; 0.11)
	2		**0.07 (0.00; 0.14)**		0.04 (–0.04; 0.11)
	3		0.06 (–0.01; 0.13)		0.04 (–0.04; 0.11)
- High versus light	1		–0.04 (–0.11; 0.03)		**–0.10 (–0.17; –0.03)**
	2		0.05 (–0.02; 0.11)		**–0.09 (–0.16; –0.01)**
	3		0.04 (–0.03; 0.10)		**–0.08 (–0.16; –0.01)**
Smoking		N = 5,666		N = 5,255	
-Former versus never	1		**–0.09 (–0.15; –0.04)**		–0.05 (–0.11; 0.01)
	2		**0.06 (0.01; 0.11)**		–0.02 (–0.08; 0.04)
	3		**0.05 (0.00; 0.11)**		–0.01 (–0.07; 0.05)
-Current versus never	1		**–0.14 (–0.22; –0.06)**		0.08 (–0.01; 0.17)
	2		**–0.15 (–0.23; –0.07)**		0.09 (–0.00; 0.17)
	3		**–0.14 (–0.22; –0.06)**		0.09 (–0.00;0.18)
Antihypertensive medication use	1	N = 5,666	**–0.33 (–0.39; –0.28)**	N = 5,255	**–0.16 (–0.21; –0.10)**
	2		–0.05 (–0.10; 0.00)		**–0.09 (–0.15; –0.03)**
	3		**–**0.03 (–0.09; 0.03)		**–0.12 (–0.19; –0.05)**
24-h ambulatory systolic blood pressure, per SD	1	N = 5,074	**–0.09 (–0.12; –0.06)**	N = 4,746	**–0.06 (–0.09; –0.03)**
	2		–0.01 (–0.04; 0.01)		–0.01 (–0.04; 0.02)
	3		–0.01 (–0.04; 0.02)		–0.01 (–0.04; 0.02)
24-h ambulatory diastolic blood pressure, per SD	1	N = 5,074	**0.09 (0.07; 0.12)**	N = 4,746	**–0.05 (–0.08; –0.02)**
	2		0.03 (–0.00; 0.05)		**–0.03 (–0.06; –0.00)**
	3		0.03 (–0.00; 0.05)		**–0.03 (–0.06; –0.00)**
Mean arterial pressure, per SD	1	N = 5,074	0.01 (–0.03; 0.03)	N = 4,746	**–0.06 (–0.09; –0.03)**
	2		0.01 (–0.02; 0.04)		–0.02 (–0.05; 0.01)
	3		0.01 (–0.02; 0.04)		–0.03 (–0.06; 0.01)
Total cholesterol, per SD	1	N = 5,664	**0.08 (0.05; 0.10)**	N = 5,255	**0.06 (0.03; 0.08)**
	2		**0.06 (0.04; 0.09)**		0.02 (–0.01; 0.05)
	3		**0.05 (0.02; 0.08)**		0.03 (–0.00; 0.06)
Waist circumference, per SD	1	N = 5,666	**–0.10 (–0.12; –0.07)**	N = 5,255	**–0.05 (–0.07; –0.02)**
	2		–0.02 (–0.05; 0.01)		0.02 (–0.01;0.05)
	3		–0.01 (–0.05; 0.02)		0.03 (–0.00;0.07)
Lower physical activity, per SD	1	N = 5,027	**–0.06 (–0.08; –0.03)**	N = 4,510	–0.02 (–0.05; 0.01)
	2		–0.01 (–0.03; 0.02)		–0.00 (–0.03; 0.03)
	3		0.01 (–0.02; 0.04)		–0.01 (–0.04; 0.02)

Standardized regression coefficients (stβ) represent the difference in retinal sensitivity or RNFL thickness (in SD) for one SD greater HbA1c, lower healthy diet score, lower cardiorespiratory fitness, for none, moderate, or high versus light total alcohol consumption, for current or former versus never smoking, for with versus without antihypertensive medication use, greater 24-h ambulatory systolic blood pressure, greater total cholesterol, greater waist circumference, or lower physical activity. One SD corresponds with 11.6 and 7.2 mm Hg for systolic and diastolic 24-h ambulatory blood pressure, respectively, 8.0 mm Hg for mean arterial pressure, 1.1 mmol/L for total cholesterol, 13.4 cm for waist circumference, 0.9% for HbA1c, 14.6 points for the healthy diet score, 0.6 W/kg for cardiorespiratory fitness, 40.9 minutes/day for physical activity, 1.6 dB for retinal sensitivity (all determined in the study population with complete data on retinal sensitivity and waist circumference), or 10.8 micrometers for RNFL thickness (RNFL thickness was determined in the study population with complete data on antihypertensive medication use [*n* = 5,666]; values per SD for other variables were numerically similar in the retinal sensitivity and RNFL thickness study populations). Bold denotes *p* < 0.05. Variables in models: Model 1: crude; Model 2: model 1 + age, sex, glucose metabolism status (where applicable), educational level; Model 3: model 2 + office systolic blood pressure (where applicable), antihypertensive medication use (where applicable), waist circumference (where applicable), total cholesterol/ HDL ratio (where applicable), lipid-lowering medication use, smoking (where applicable), and alcohol consumption (where applicable; a precise overview of covariates entered per model [for all analyses] is presented in the Methods). RNFL, retinal nerve fiber layer; SD, standard deviation; CI, confidence interval; HbA1c, glycated hemoglobin A1c; HDL, high density lipoprotein.

### Healthy diet score without alcohol consumption

After full adjustment (model 3), lower healthy diet score was significantly associated with lower retinal sensitivity and lower RNFL thickness (per SD, standardized beta [95% CI], –0.06 [–0.09; –0.03], and –0.03 [–0.06; –0.00], respectively).

### Cardiorespiratory fitness

After full adjustment (model 3), lower cardiorespiratory fitness was significantly associated with lower retinal sensitivity, but not with lower RNFL thickness (per SD, standardized beta [95% CI], –0.05 [–0.08; –0.01], and –0.03 [–0.07; 0.01], respectively).

### Alcohol consumption

After full adjustment (model 3), high versus light alcohol consumption was not associated with lower retinal sensitivity but was significantly associated with lower RNFL thickness (standardized beta [95% CI], 0.04 [–0.03; 0.10], and –0.08 [–0.16; –0.01], respectively).

### Smoking

After full adjustment (model 3), current versus never smoking was significantly associated with lower retinal sensitivity but was not associated with RNFL thickness (standardized beta [95% CI], –0.14 [–0.22; –0.06], and 0.09 [–0.00; 0.18], respectively).

In contrast, after full adjustment (model 3), former versus never smoking was significantly associated with greater retinal sensitivity and was not associated with lower RNFL thickness (standardized beta [95% CI], 0.05 [0.00; 0.11], and –0.01 [–0.07; 0.05], respectively).

### Blood pressure

After full adjustment (model 3), antihypertensive medication use was not associated with retinal sensitivity, but was significantly associated with lower RNFL thickness (per SD, standardized beta [95% CI], –0.03 [–0.09; 0.03], and –0.12 [–0.19; –0.05], respectively).

After full adjustment (model 3), greater 24-h ambulatory systolic, diastolic, and mean arterial blood pressure were not associated with retinal sensitivity (per SD, standardized beta [95% CI], –0.01 [–0.04; 0.02], 0.03 [–0.00; 0.05], and 0.01 [–0.02; 0.04], respectively).

After full adjustment (model 3), greater 24-h ambulatory diastolic, but not 24-h ambulatory systolic blood pressure or mean arterial blood pressure, was significantly associated with lower RNFL thickness (per SD, standardized beta [95% CI], –0.03 [–0.06; –0.00], –0.01 [–0.04; 0.02], and –0.03 [–0.06; 0.01], respectively).

### Cholesterol

After full adjustment (model 3), greater total cholesterol was significantly associated with *greater* retinal sensitivity but was not associated with RNFL thickness (per SD, standardized beta [95% CI], 0.05 [0.02; 0.08], and 0.03 [–0.00; 0.06],respectively).

### Waist circumference

After full adjustment (model 3), greater waist circumference was neither associated with retinal sensitivity, nor with RNFL thickness (per SD, standardized beta [95% CI], –0.01 [–0.05; 0.02], and 0.03 [–0.00; 0.07], respectively).

### Physical activity

After full adjustment (model 3), lower physical activity was neither associated with retinal sensitivity, nor with RNFL thickness (per SD, standardized beta [95% CI], 0.01 [–0.02; 0.04], and –0.01 [–0.04; 0.02], respectively).

### Tests for interaction and stratified analyses

Sex did not modify any of the associations, but glucose metabolism status did (all *p*-values for interaction are shown in Supplementary Table 4). Type 2 diabetes modified the association of 24-h ambulatory systolic blood pressure with retinal sensitivity (p_interaction_ < 0.001); the association of 24-h ambulatory systolic blood pressure with RNFL thickness (p_interaction_ = 0.049); and the association of total cholesterol with RNFL thickness (p_interaction_ = 0.04). Additionally, prediabetes inconsistently modified the associations of healthy diet score and physical activity with retinal sensitivity.

In individuals with, but not without, type 2 diabetes, greater 24-h ambulatory systolic blood pressure was significantly associated with lower retinal sensitivity, but not with lower RNFL thickness (model 3; in individuals with type 2 diabetes, per SD, standardized beta [95% CI], –0.06 [–0.12; –0.04], and –0.06 [–0.13; 0.00], respectively; Supplementary Table 5). In addition, we observed a similar pattern for 24-h ambulatory diastolic and mean arterial blood pressure. Then, in individuals with, but not without, type 2 diabetes, greater total cholesterol was significantly associated with greater RNFL thickness (model 3; in individuals with type 2 diabetes, per SD, standardized beta [95% CI], 0.09 [0.03; 0.16]). However, we did not observe this pattern for retinal sensitivity.

### Additional analyses

We generally observed quantitatively similar results in a range of additional analyses (all results are reported in the Supplementary Results section and are shown in Supplementary Tables 6–12). Here we highlight one main finding. After full adjustment (model 3), greater age was associated with lower retinal sensitivity and lower RNFL thickness (standardized beta [95% confidence interval], per year –0.04 [–0.05; –0.04] and –0.01 [–0.01; –0.01], respectively; Supplementary Table 8). Hence, the beta for 1 SD greater HbA1c corresponds with approximately 1.3 year of aging for retinal sensitivity and 5.0 years of aging for RNFL thickness; the beta of 1 SD lower adherence to a healthy diet corresponds with approximately 1.5 years of aging for retinal sensitivity and 3.0 years of aging for RNFL thickness; and the beta of 1 SD lower cardiorespiratory fitness corresponds with approximately 1.3 year of aging for retinal sensitivity and 3.0 years of aging for RNFL thickness. Therefore, added up, the combination of these three adverse factors corresponds with approximately 4.1 and 11.0 years of “aging” for, respectively, retinal sensitivity and RNFL thickness.

## DISCUSSION

The present population-based study has four main findings. First, we found significant associations with lower retinal sensitivity of greater HbA1c, lower healthy diet score, lower cardiorespiratory fitness, current versus never smoking, and greater 24-h ambulatory blood pressure, though the latter only in individuals with, but not without, type 2 diabetes. Second, we found significant associations with lower RNFL thickness of greater HbA1c, lower healthy diet score, lower cardiorespiratory fitness, high versus light alcohol consumption, current versus never smoking, and antihypertensive medication use. Third, greater total cholesterol was associated with *greater* retinal sensitivity and *greater* RNFL thickness, though the latter only in individuals with, but not without, type 2 diabetes. Fourth, waist circumference and physical activity were not associated with outcomes under study.

Our findings on RNFL thickness are in line with findings from most previous population-based studies [22–32]. Importantly, the present study is the first population-based study to investigate the associations of potentially modifiable risk factors for dementia with retinal sensitivity [21]. In addition, the present study is the first study to report associations of 24-h ambulatory-assessed blood pressure levels, waist circumference, adherence to a healthy diet, cardiorespiratory fitness, and accelerometer-assessed physical activity with RNFL thickness.

Mechanistically, most risk factors that were significantly associated with outcomes can increase levels of oxidative stress in the retina, which can lead to retinal microvascular dysfunction, loss of retinal neural structures (including RNFL thinning), and retinal dysfunction (i.e., resulting in lower retinal sensitivity) [2, 43]. Additionally, antihypertensive medication use, lower cardiorespiratory fitness and a less healthy diet may be associated with more retinal neurodegenerative changes as they, respectively, reflect past exposure to higher levels of blood pressure over time (i.e., hypertension), lower exposure to neuroprotective factors in the retina [44]; and lower exposure to certain nutrients which can reduce levels of oxidative stress [45]. Biologically, impaired microvascular function likely predisposes the retinal capillaries to a high intracapillary pressure, which is detrimental for the capillaries and can lead to ischemia and a hampered clearance of toxins from the neuronal tissue, both of which can activate pathways that lead to neurodegeneration [2, 43]. Physiologically, up to 20–50% of retinal ganglion cells can be lost before worse retinal function is detectable [46]. In addition, progressive loss of neural structures can also increasingly predispose retinal neurons to ischemia as neuronal structures contribute to function of the neurovascular coupling unit, which tightly regulates the supply of nutrients (e.g., oxygen) to retinal neurons [1, 2, 43].

In participants with, but not without, type 2 diabetes greater 24-h ambulatory systolic blood pressure was significantly associated with lower retinal sensitivity and numerically similar in strength, though not statistically significant, with lower RNFL thickness. Biologically, as hyperglycemia is detrimental for neuronal and microvascular structures, which regulate capillary pressure, individuals with, versus without, type 2 diabetes may be more susceptible to hypertension [43].

Directionally inconsistent with our hypothesis, *greater* total cholesterol was associated with *greater* retinal sensitivity and *greater* RNFL thickness, though the latter only in individuals with, but not without, type 2 diabetes. Mechanistically, a greater total cholesterol level, which reflects higher levels of circulating cholesterol, may be beneficial for the retina as cholesterol is an important contributor to the formation of neuronal synapses [47]. Then, as under hyperglycemic circumstances neuronal and microvascular structures (i.e., neurovascular coupling unit) are functionally impaired, the ability to ensure a continuous supply of cholesterol to the retina is likely reduced, resulting in lower levels of retinal cholesterol, and subsequently less synaptogenesis, in individuals with, versus without, type 2 diabetes [47].

Waist circumference was not associated with outcomes, possibly because the effects of adiposity on neural retina tissue are bidirectional. Mechanistically, higher levels of visceral (white) adipose tissue, which greater waist circumference represents, likely leads to higher levels of adipokines in the circulation, which may have both neuroprotective as well as neurodegenerative effects on retinal neurons [48]. For example, directionally opposing effects have been reported for tumor necrosis factor-alpha [TNF-alpha]), a relatively well-studied adipokine [48].

Lower physical activity was not associated with outcomes under study, possibly because other factors than the amount of physical activity determine the extent of the neuroprotective physiological response to physical activity [49]. Indeed, besides the amount of physical activity, also genetic factors and the type, frequency, and intensity of activity have been found to determine the physiological response to physical activity [49].

Our findings contribute to the increasing evidence that there may be an opportunity to improve clinical care via the use of retinal neural biomarkers. First, our findings are consistent with the concept that retinal sensitivity and RNFL thickness may be biomarkers for the monitoring of therapeutic strategies that aim to prevent cerebral neurodegeneration in the absence of clinical dementia [16, 20]. However, in order to able to move from a research setting to a clinical setting, future trials are warranted to further investigate whether modification of potentially modifiable risk factors for dementia can actually lead to a detectable increase in retinal sensitivity and RNFL thickness [50]. Second, the modification of a combination of risk factors may result in a considerable reduction of retinal neuronal aging. Indeed, added up, 1 SD greater HbA1c, 1 SD lower adherence to a healthy diet, and 1 SD lower cardiorespiratory fitness correspond with approximately 4.1 and 11.0 years of “aging” for, respectively, retinal sensitivity and RNFL thickness.

Strengths of this study are 1) the large size of this population-based cohort with oversampling of individuals with type 2 diabetes, which enabled accurate comparison of individuals with and without diabetes; 2) the extensive number of potential confounders that were considered; 3) the use of state-of-the-art methods to assess all variables included in thisstudy [50].

The study has certain limitations. First, due to the cross-sectional nature of the study, causal inferences should be made with caution [50]. Mechanistically, hyperglycemia and hypertension may lead to neurodegeneration but the reverse may also be true, i.e., there may be a vicious cycle [43]. Intact neurovascular interaction is required for normal microvascular function and impaired microvascular function may aggravate hyperglycemia and hypertension [43]. Second, we may have underestimated the strength of the associations under study if such associations were stronger in participants that were not included in the study population (who generally tended to be less healthy) [50]. Third, although we took an extensive set of confounders into account, we cannot fully exclude bias due to unmeasured confounding (e.g., due to environmental factors such as air pollution) [50]. Fourth, we may have underestimated the strength of certain associations under study (e.g., diet) as we adjusted for certain variables which may (in part) reflect the effects on the outcomes of certain potentially modifiable risk factors for dementia (e.g., we adjusted for glucose metabolism status and blood pressure; and a healthy dietary intake may in part protect against neurodegeneration via reducing the risk of hyperglycemia and hypertension) [50]. Last, we studied Caucasian individuals aged 40–75 years and therefore our results may be generalizable to such a population; whether these results also apply to other populations requires furtherstudy [50].

In summary, the present population-based study demonstrated that most potentially modifiable risk factors for dementia were independently associated with indices of retinal neuronal function (i.e., retinal sensitivity) and structure (i.e., RNFL thickness). Hence, retinal indices of neural function and structure may be biomarkers for the monitoring of therapeutic strategies that aim to prevent early-stage cerebral neurodegeneration and, ultimately, dementia.

## FUNDING

This study was supported by the European Regional Development Fund via OP-Zuid, the Province of Limburg, the Dutch Ministry of Economic Affairs (grant 31O.041), Stichting De Weijerhorst (Maastricht, the Netherlands), the Pearl String Initiative Diabetes (Amsterdam, the Netherlands), the Cardiovascular Centre (CVC, Maastricht, the Netherlands), CARIM School for Cardiovascular Diseases (Maastricht, the Netherlands), CAPHRI School for Public Health and Primary Care (Maastricht, the Netherlands), NUTRIM School for Nutrition and Translational Research in Metabolism (Maastricht, the Netherlands), Stichting Annadal (Maastricht, the Netherlands), Health Foundation Limburg (Maastricht, the Netherlands), Perimed (Järfälla, Sweden), Diabetesfonds grant 2016.22.1878 (Amersfoort, The Netherlands), Oogfonds (Utrecht, The Netherlands) and by unrestricted grants from Heidelberg Engineering (Heidelberg, Germany), Janssen-Cilag B.V. (Tilburg, the Netherlands), Novo Nordisk Farma B.V. (Alphen aan den Rijn, the Netherlands), and Sanofi-Aventis Netherlands B.V. (Gouda, the Netherlands).

## CONFLICT OF INTEREST

The authors have no conflict of interest to report.

## DATA AVAILABILITY

Data are available from The Maastricht Study for any researcher who meets the criteria for access to confidential data; the corresponding author may be contacted to request data.

## Supplementary Material

Supplementary Material
