# Supplementary material for: Retinal Functional and Structural Neural Indices: Potential Biomarkers for the Monitoring of Cerebral Neurodegeneration: The Maastricht Study
Source: J Alzheimers Dis. 2023 Jun 13;93(4):1471–83. doi: 10.3233/JAD-230104 (PMC10357177; doi:10.3233/JAD-230104)
Supplement: Supplementary Material [file jad-93-jad230104-s001.pdf]

Supplementary Material

Retinal Functional and Structural Neural Indices: Potential Biomarkers for the Monitoring of Cerebral Neurodegeneration: The Maastricht Study

SUPPLEMENTARY METHODS

Study population and design

We used data from The Maastricht Study, a prospectively designed, population-based observational cohort study. The rationale and methodology have been described previously [1]. In brief, the study focuses on the etiology, pathophysiology, complications, and comorbidities of type 2 diabetes mellitus and is characterized by an extensive phenotyping approach. Eligible for participation were all individuals aged between 40 and 75 years and living in the southern part of the Netherlands. Participants were recruited through mass media campaigns and from the municipal registries and the regional Diabetes Patient Registry via mailings. Recruitment was stratified according to known type 2 diabetes status, with an oversampling of individuals with type 2 diabetes, for reasons of efficiency [1]. The present report includes cross-sectional data of 7,689 participants who completed the baseline survey between November 2010 and December 2017. The examinations of each participant were performed within a time window of three months. The study has been approved by the institutional medical ethical committee (NL31329.068.10) and the Minister of Health, Welfare and Sports of the Netherlands (Permit 131088-105234-PG). All participants gave written informed consent [1].

Retinal sensitivity

We used the Heidelberg Edge Perimeter (Heidelberg Engineering, Heidelberg, Germany), a static flicker perimeter, to assess retinal sensitivity [2]. Measurements were performed in a dimly lit room under supervision of a trained examiner. Any refractive error was corrected for with external lenses. For each eye, retinal sensitivity was measured at 54 coordinates in the central and peri macular area (between 48° in the transverse plane and 42° in the sagittal plane) and results were averaged in to “retinal sensitivity”. In brief, the participant was instructed to fixate their vision on a focus point and to indicate when they observed a static white light stimulus by pressing a joystick button. Light stimuli varying in strength between 0 and 35 decibel (dB) and sized 0.43° in diameter (Goldmann perimeter size III) were presented on an isoluminant background of 10

candela per square meter. To, per coordinate, determine the threshold of visual perception (i.e., the threshold at which the weakest presented visual stimulus could be perceived), we used the adaptive staircase thresholding algorithm standard automated perimetry 24-2 pattern setting. The intra-observer reliability for the assessment of the retinal sensitivity is 0.95 [3].

The device automatically calculated the following indices of measurement quality: the percentage of false positive entries, the percentage of false negative entries, and the number of fixation errors. A false positive entry indicates that the participant responded when no stimulus was presented [4]. A false negative entry indicates that the participant did not respond to a stimulus that should be visible based on an earlier response [4]. A fixation error indicates that the fixation of the eye deviated more than 5° from the central fixation point [4]. We defined sufficient measurement quality as ≤ 15% false positive responses and ≤ 30% false negative responses [4]. To reduce measurement error, we averaged retinal sensitivity of both eyes when data of sufficient quality from both eyes were available (n=5,087 participants [89.8%]). When data from only one eye were available (n=579 participants [10.2%]) we used the retinal sensitivity of that eye in the analyses. More details are provided in the Supplementary Methods.

RNFL thickness

We assessed peripapillary RNFL thickness (μm) in both eyes using optical coherence tomography (OCT; Spectralis unit and Eye Explorer version 5.7.5.0 software; Heidelberg Engineering, Heidelberg, Germany; 3.45-mm-diameter-circle scan, manually centered on the optic nerve head, 12°, 768 voxels, 100 automatic real-time tracking). Intra- and interindividual reliability, expressed as intraclass correlation coefficients, are 0.97 and 0.96, respectively. At least 15 min before the examination pupils were dilated with topical 0.5% tropicamide and 2.5% phenylephrine. Experienced graders masked to clinical information on the participants reviewed the OCT scans and graded their quality. OCT images were excluded if one of the following criteria was present: scan error (i.e., incomplete scan, poor centering of the circular scan on the optic nerve head, RNFL layer incorrectly defined, or technical problem with the OCT device) or poor imaging quality (signal-to-noise ratio<15 dB) [5]. If data from both eyes were available (n=2,711 participants) we averaged RNFL thickness of both eyes in order to reduce measurement error. If data from only one eye were available (n=2,544 participants), we used the RNFL thickness of that

eye in the analyses. More details, including on quality criteria, are shown in the Supplementary Methods.

Grading of OCT circle scans

OCT scans were considered of sufficient quality if all the following criteria were met: good centering of the circular scan on the optic nerve head (examples of good, poor and very poor centering are shown in Supplementary Figure 1); complete (data of all 768 voxels was available); automatic quality ≥15 dB (an example of a scan with poor quality imaging is shown in Supplementary Figure 2); and no measurement error present (examples of all assessed measurement errors are shown in Supplementary Figure 2). The percentage of agreement for selection of scans with sufficient quality ranged between 90% and 94% for four trained graders and was 70% for one grader (n=50 OCT scans per comparison).

Time lag between assessment of retinal sensitivity or RNFL thickness and other covariates

For a subset of participants retinal sensitivity and RNFL thickness measurements were performed as part of a catch-up visit (n=227 and n=305, respectively). We checked whether exclusion of these participants or additional adjustment for the lag time between measurements altered associations under study and this was not the case (data not shown).

Healthy diet and alcohol consumption

We assessed dietary intake, including alcohol consumption, with a validated food frequency questionnaire [6], and calculated the Dutch Healthy Diet index sum score, a measure of adherence to the Dutch dietary guidelines 2015 [6, 7]. The Dutch Healthy diet index sum score was developed based on 15 components of a Dutch diet, however as data on coffee intake (one of the 15 items) were presently not available, for this study we calculated the Dutch Healthy Diet index sum score based on 14 components (i.e., all 15 components except coffee intake). Next, as we investigated alcohol consumption separately from other components of a healthy diet, we recalculated the Dutch Healthy diet index sum score so that alcohol consumption was left out of this total score (therefore the Dutch Healthy diet index sum score used in the main analyses to study healthy diet as a main determinant consisted out of 13 components in total).

We categorized alcohol consumption into none (<1 unit/week [for both men and women]), light (≥1 unit/ week to 1 unit/day for men, ≥1 unit/ week to 0.5 unit/day for women), moderate (>1 to 2 units/day for men, >0.5 to 1 unit/day for women), and high (>2 units/day for men, >1 units/day for women) where 1 unit was defined as 10 gram/day (g/d) of total alcohol (i.e., ethanol) consumption, as previously described [8]. In analyses we used light drinkers as a reference group because we cannot distinguish so-called sick quitters from never drinkers (i.e., life-long abstainers) within the none consumers [9].

In analyses with categories of alcohol consumption we used light drinkers as a reference group because we cannot distinguish so-called sick quitters from never drinkers (i.e., life-long abstainers) within the none consumers [9].

We also assessed alcohol consumption with a different questionnaire than the validated food frequency questionnaire [1]. We used data on alcohol consumption from this different questionnaire in analyses where alcohol consumption was not the main determinant because data from this questionnaire were available in a larger number of participants (i.e., n=5,666 instead of n=5,377). For analyses where alcohol consumption was not the main determinant, we categorized alcohol consumption as none (for women and men, 0 units/week), moderate (for women and men, respectively, 1-7 units and 1-14 units of alcohol consumption/week), and high (for women and men, respectively, >7 units and >14 units of alcohol consumption/week). We did not calculate a light alcohol consumption group because this questionnaire did not allow to distinguish between light and moderate alcohol consumption.

Cardiorespiratory fitness

We assessed cardiorespiratory fitness from a graded submaximal exercise protocol performed on a cycle ergometer system (CASE^{TM} version 6.6 in combination with e-bike; GE Healthcare, Milwaukee, WI), as described previously [10]. Cardiorespiratory fitness was defined as the maximum power output W_{max} and was adjusted for body mass (i.e., $W_{max} \cdot kg^{-1}$). During the submaximal exercise protocol blood pressure and electrical activity of the heart rhythm were monitored as described previously. Participants were excluded from the submaximal cycle ergometer test if they had experienced cardiovascular complications in the preceding three months; had an abnormal resting electrocardiogram; had a medical history of certain cardiovascular complications (e.g., pericarditis or hypertrophic cardiomyopathy); had severe hypertension

(systolic blood pressure ≥180 mmHg and/or diastolic blood pressure ≥110 mm Hg); had a history of kidney failure; or had an implantable cardioverter-defibrillator or a pacemaker.

The protocol consisted of a short warm-up period and at most seven stages with increasing workload. Participants were instructed to cycle at a cadence of 60–70 rotation per minute (rpm) during a short familiarization period without any external workload. For the first exercise stage, external workload was set at 25 W. Then, every 2 min the external workload was consecutively increased with 25 W. At the end of each stage, heart rate and blood pressure were measured. Further, the participant was asked to provide a rating of perceived exertion based on the 15-point Borg-scale, which is an interval scale that ranges from 6 points ("no exertion at all") up to 20 points ("maximal exertion"). The exercise protocol was considered as "completed" when heart rate reached ≥85% of the estimated maximum heart rate (220 minus the age) or when a rating of perceived exertion ≥ 17 was scored by the participant. Then, the test was also terminated by the end of stage 7 (workload of 175 W) if the heart rate was < 85% and rating of perceived exertion was < 17); or (prematurely) for medical reasons or when the participant was unwilling to continue. More details can be found elsewhere [10].

Blood pressure

We assessed 24-h ambulatory blood pressure (mm Hg) with an oscillometric device (WatchBP O3; Microlife, Widnau, Switzerland) [11]. We assessed antihypertensive medication use, an index of past exposure to relatively higher levels of blood pressure, via an interview.

Cholesterol

We determined total cholesterol (mmol/L) in fasting venous plasma sample [1].

Physical activity

We measured daily activity levels (h/day) with the activPAL3^{TM} physical activity monitor (PAL technologies, Glasgow, UK), as previously described [12]. Participants were asked to wear the accelerometer for 8 consecutive days, without removing the device at any time. The total amount of physical activity (stepping time) was based on the stepping posture and calculated as the mean time (min) spent stepping during waking time per day, where standing time was not included. The method used to determine waking time has been previously described [12].

Covariates

As described previously [1], we assessed educational level (low, intermediate, high), socio-economic status (income level and occupational status; both presently available only in a subset of participants) [13], history of cardiovascular disease, mobility limitation, and age-related macular degeneration by questionnaire [1]; high-density lipoprotein (HDL), low-density lipoprotein (LDL), triglycerides, fasting plasma glucose, and serum creatinine in fasting venous blood samples [1]; assessed glucose metabolism status based on fasting plasma glucose and oral glucose tolerance test-derived 2-h post load glucose [1]; assessed office blood pressure and body mass index as part of a physical examination [1]; and current presence of cataract and medication use as part of an interview; calculated the estimated glomerular filtration rate (eGFR) based on serum creatinine only [14], since cystatin C was not presently available in all study participants; measured urinary albumin excretion in two 24-h urine collections; assessed presence of retinopathy in both eyes via fundus photography; and used an automated refractor and noncontact tonometer (Tonoref II; Nidek, Gamagordi, Japan) to assess spherical equivalent and intraocular pressure in both eyes. Glaucoma was defined as use of intraocular pressure-lowering medication, intraocular pressure higher than 21 mm Hg in any eye (91.3% of all participants had data on intraocular pressure available for at least 1 eye), or both. Spherical equivalent was defined as the mean spherical equivalent of both eyes (available for 91.1% of all participants) or as the spherical equivalent of the eye for which data were available.

Statistical analyses

Collinearity diagnostics (i.e., tolerance <0.10 and/or variance inflation factor >10) were used to detect multicollinearity between covariates.

Additional analyses

To assess the robustness of our findings we performed a range of additional analyses. First, we repeated the analyses with additional adjustment for lifestyle factors (dietary intake, physical activity). Adjustment for these potential confounders was not included in the main analyses because data were missing for a relatively large number of participants (up to n=904 had missing data on one or more of these variables). Second, we studied the associations of office systolic and

diastolic blood pressure with the outcomes under study; and associations of mean arterial pressure, estimated from office blood pressure (using the same formula as for 24-h ambulatory blood pressure), with the outcomes under study. We did not use these covariates in the main analyses because they are less precise estimates of blood pressure than 24-h ambulatory blood pressure [15, 16]. Third, we studied the association of lipid-lowering medication with outcomes under study. Fourth, we performed additional analyses in which we omitted antihypertensive medication use from the model for associations of 24-h ambulatory systolic, diastolic, and mean arterial pressure with the outcomes under study to investigate how strongly antihypertensive medication use affected these associations. In addition, to investigate how strongly office systolic blood pressure affected the association of antihypertensive medication use with the outcomes under study, we performed additional analyses without adjustment for office systolic blood pressure. Fifth, to obtain a more detailed insight into how lipids are associated with outcomes under study, we studied associations of individual types of lipids (i.e., HDL, LDL, triglycerides) with outcomes under study (all three covariates were entered in the same model). Sixth, we studied the associations of body-mass index with outcomes under study. We did not use body-mass index in the main analyses because waist circumference is a more precise measure of visceral fat than body mass index [17]. Seventh, to more robustly investigate whether hyperglycemia may be a determinant of outcomes under study, we investigated whether fasting plasma glucose and 2-h post load glucose were associated with retinal sensitivity and RNFL thickness. Eighth, to check whether both alcohol questionnaires that we used were similar, we studied the associations of alcohol consumption with the outcomes under study using data from both questionnaires. For this comparison only, we categorized alcohol consumption assessed with the food frequency questionnaire as none, moderate, and high alcohol consumption. Nineth, as non-linear associations of alcohol intake with cerebral neural measures have previously been described [18], we tested for a quadratic association by entering alcohol consumption (continuous variable) and a quadratic term for alcohol consumption in the model (i.e., alcohol consumption[continuous variable]*alcohol consumption[continuous variable]; we used the formula $y=x^2+x$). If the p-value of the quadratic term was <0.05, the association was considered to be statistically better described by a non-linear, quadratic association than by a linear association [19]. Tenth, we re-analyzed the associations of current and never smoking with outcomes under study and used former smoking as a reference group. Eleventh, and only for cardiorespiratory fitness and physical activity, we adjusted

associations for mobility limitation. We entered mobility limitation into a separate model because adjustment for this covariate may be overadjustment (i.e., mobility limitation is strongly associated with cardiorespiratory fitness and physical activity but the association with retinal sensitivity and RNFL thickness is less clear) [20]. Twelfth, we additionally adjusted for kidney variables (eGFR and urinary albumin excretion), history of cardiovascular disease, intraocular pressure, spherical equivalent, cataract, retinopathy, glaucoma, and age-related macular degeneration. We adjusted for these covariates in separate models because they may be confounders but may also (in part) be mediators or descendants of the outcome [20]. Thirteenth, we performed additional analyses in which we excluded participants with either retinopathy, cataract, age-related macular degeneration, glaucoma, or any combination of these morbidities. Fourteenth, we repeated the analyses applying more strict quality criteria (i.e., when we in addition to the other criteria excluded measurements with >20% fixation errors entries). We did not include individuals with >20% fixation errors in the main analyses as this would have led to a strong reduction in the size of the study population (up to n=2,254 would not have been included in the analyses). Fifteenth, we replaced educational status with occupational status or income level; and we replaced glucose metabolism status with fasting plasma glucose, 2-h post load glucose, or HbA1c. Sixteenth, to test robustness of interaction findings for glucose metabolism status we performed tests of interactions with continuous measures of glycemia (i.e., fasting plasma glucose, 2-h post load glucose, or HbA1c) if associations were modified by glucose metabolism status. Last, we studied the associations of age with retinal sensitivity and RNFL thickness. We performed these analyses so that we could compare with how many years of "aging" the betas for determinants under study correspond.

REFERENCES

[1] Schram MT, Sep SJ, van der Kallen CJ, Dagnelie PC, Koster A, Schaper N, Henry RM, Stehouwer CD (2014) The Maastricht Study: an extensive phenotyping study on determinants of type 2 diabetes, its complications and its comorbidities. *Eur J Epidemiol* **29**, 439-451.

[2] Kaczorowski K, Mulak M, Szumny D, Misiuk-Hojło M (2015) Heidelberg Edge Perimeter: the new method of perimetry. *Adv Clin Exp Med* **24**, 1105-1112.

[3] Cui Q, Gogt P, Lam J, Siraj S, Hark L, Myers J, Katz L, Waisbourd M (2019) Validation and reproducibility of the Heidelberg Edge Perimeter in the detection of glaucomatous visual field defects. *Int J Ophthalmol* **12**, 577-581.

[4] Heidelberg Engineering GmbH Germany (2014) *Heidelberg Engineering User Manual. Software Version 3.1*.

[5] De Clerck EEB, Schouten JSAG, Berendschot TTJM, Beckers HJM, Schaper NC, Schram MT, Stehouwer CDA, Webers CAB (2017) Loss of temporal peripapillary retinal nerve fibers in prediabetes or type 2 diabetes without diabetic retinopathy: The Maastricht Study. *Invest Ophthalmol Vis Sci* **58**, 1017-1027.

[6] van Dongen MC, Wijckmans-Duysens NEG, den Biggelaar LJ, Ocke MC, Meijboom S, Brants HA, de Vries JH, Feskens EJ, Bueno-de-Mesquita HB, Geelen A, Stehouwer CD, Dagnelie PC, Eussen SJ (2019) The Maastricht FFQ: Development and validation of a comprehensive food frequency questionnaire for the Maastricht study. *Nutrition* **62**, 39-46.

[7] Looman M, Feskens EJ, de Rijk M, Meijboom S, Biesbroek S, Temme EH, de Vries J, Geelen A (2017) Development and evaluation of the Dutch Healthy Diet index 2015. *Public Health Nutr* **20**, 2289-2299.

[8] Davis BJ, Vidal JS, Garcia M, Aspelund T, van Buchem MA, Jonsdottir MK, Sigurdsson S, Harris TB, Gudnason V, Launer LJ (2014) The alcohol paradox: light-to-moderate alcohol consumption, cognitive function, and brain volume. *J Gerontol A Biol Sci Med Sci* **69**, 1528-1535.

[9] Tsubono Y, Yamada S, Nishino Y, Tsuji I, Hisamichi S (2001) Choice of comparison group in assessing the health effects of moderate alcohol consumption. *JAMA* **286**, 1177-1178.

[10] JHPM VDV, Koster A, JD VDB, Sep SJS, CJH VDK, Dagnelie PC, Schram MT, Henry RMA, Eussen S, MCJM VAND, Stehouwer CDA, Schaper NC, Savelberg H (2017) Sedentary behavior, physical activity, and fitness-The Maastricht Study. *Med Sci Sports Exerc* **49**, 1583-1591.

[11] Zhou TL, Kroon AA, Reesink KD, Schram MT, Koster A, Schaper NC, Dagnelie PC, van der Kallen CJH, Sep SJS, Stehouwer CDA, Henry RMA (2018) Blood pressure variability in individuals with and without (pre)diabetes: The Maastricht Study. *J Hypertens* **36**, 259-267.

[12] van der Berg JD, Willems PJ, van der Velde JH, Savelberg HH, Schaper NC, Schram MT, Sep SJ, Dagnelie PC, Bosma H, Stehouwer CD, Koster A (2016) Identifying waking time in 24-h accelerometry data in adults using an automated algorithm. *J Sports Sci* **34**, 1867-1873.

[13] Qi Y, Koster A, van Boxtel M, Kohler S, Schram M, Schaper N, Stehouwer C, Bosma H (2019) Adulthood socioeconomic position and type 2 diabetes mellitus-a comparison of education, occupation, income, and material deprivation: The Maastricht Study. *Int J Environ Res Public Health* **16**, 1435.

[14] Shahbaz H, Gupta M (2021) Creatinine clearance. In *StatPearls*, Treasure Island (FL).

[15] Hutcheon JA, Chiolero A, Hanley JA (2010) Random measurement error and regression dilution bias. *BMJ* **340**, c2289.

[16] Myers MG, Kaczorowski J (2017) Office blood pressure is lower than awake ambulatory blood pressure at lower targets for treatment. *J Clin Hypertens (Greenwich)* **19**, 1210-1213.

[17] Ross R, Neeland IJ, Yamashita S, Shai I, Seidell J, Magni P, Santos RD, Arsenault B, Cuevas A, Hu FB, Griffin BA, Zambon A, Barter P, Fruchart JC, Eckel RH, Matsuzawa Y, Despres JP (2020) Waist circumference as a vital sign in clinical practice: a Consensus Statement from the IAS and ICCR Working Group on Visceral Obesity. *Nat Rev Endocrinol* **16**, 177-189.

[18] McEvoy LK, Fennema-Notestine C, Elman JA, Eyler LT, Franz CE, Hagler DJ, Jr., Hatton SN, Lyons MJ, Panizzon MS, Dale AM, Kremen WS (2018) Alcohol intake and brain white matter in middle aged men: Microscopic and macroscopic differences. *Neuroimage Clin* **18**, 390-398.

[19] Goel S, Sharma A, Garg A (2018) Effect of alcohol consumption on cardiovascular health. *Curr Cardiol Rep* **20**, 19.

[20] Schisterman EF, Cole SR, Platt RW (2009) Overadjustment bias and unnecessary adjustment in epidemiologic studies. *Epidemiology* **20**, 488-495.

SUPPLEMENTARY RESULTS

We generally observed quantitatively similar results in a range of additional analyses. First, we had numerically similar results after we additionally adjusted associations under study for lifestyle factors (i.e., dietary intake and physical activity; Supplementary Tables 6 and 7). Second, systolic, diastolic, and mean arterial blood pressure, estimated from office blood pressure measurements, were associated with greater retinal sensitivity and with lower RNFL thickness (Supplementary Table 8). Third, after full adjustment (model 3), lipid-lowering medication use was significantly associated with lower retinal sensitivity but was not associated with RNFL thickness (per SD, standardized beta [95% CI], -0.08 [-0.14; -0.01], and 0.05 [-0.02; 0.13], respectively; Supplementary Table 8). Fourth, associations of systolic, diastolic, and mean arterial blood pressure (estimated from 24-h ambulatory blood pressure measurements) with outcomes under study did not materially change when we omitted antihypertensive medication use from the model (Supplementary Table 8). Similarly, associations of antihypertensive medication use with outcomes remained similar when we did not adjust for office systolic blood pressure (Supplementary Table 8). Fifth, greater HDL and LDL, but not triglycerides, were associated with greater retinal sensitivity and greater RNFL thickness (Supplementary Table 8). Sixth, greater body-mass index was associated with greater RNFL thickness, but not with retinal sensitivity (Supplementary Table 8). Seventh, fasting plasma glucose and 2-h post load glucose were associated with lower retinal sensitivity and lower RNFL thickness (Supplementary Table 8). Eighth, we had similar findings when we studied the associations of alcohol consumption with the outcomes under study using data from both questionnaires (both questionnaires are explained in the Supplementary Methods; Supplementary Table 8). Ninth, the association of alcohol consumption with retinal sensitivity was non-linear (i.e., dome shaped; $p_{quadratic}<0.01$) and the association of alcohol consumption with RNFL thickness was linear ($p_{quadratic}>0.05$; Supplementary Figure 3). Tenth, current versus former smoking, and, less strongly, never versus former smoking were associated with lower retinal sensitivity and greater RNFL thickness (Supplementary Table 8). Eleventh, we had numerically similar findings when we additionally adjusted associations of lower cardiorespiratory fitness and lower physical activity with outcomes under study for mobility limitation (Supplementary Table 8). Twelfth, results did generally not materially change after additional adjustment for kidney variables (eGFR and urinary albumin excretion), history of cardiovascular disease, intraocular pressure, spherical equivalent, cataract,

retinopathy, glaucoma, and age-related macular degeneration (Supplementary Tables 6 and 7). There was one exception, when we additionally adjusted for spherical equivalent, the association of current versus never smoking with RNFL thickness was strongly attenuated (when we adjusted for intraocular pressure instead of both intraocular pressure and spherical equivalent [which is shown in Supplementary Table 7] we did not observe this strong attenuation). Thirteenth, results were numerically similar after exclusion of participants with cataract, retinopathy, glaucoma, and age-related macular degeneration, except for the association between total cholesterol and RNFL thickness which was strongly attenuated after exclusion of these participants (Supplementary Table 9). Fourteenth, we had similar findings when we applied more strict quality criteria for analyses in which retinal sensitivity was the outcome (Supplementary Table 9). Fifteenth, we had similar findings when we replaced educational status with occupational status or income level; and when we replaced glucose metabolism status with fasting plasma glucose, 2-h post load glucose, or HbA1c (Supplementary Tables 10 and 11). There were three inconsistent exceptions: when we replaced educational level with occupational level, we found that the association of waist circumference with retinal sensitivity became stronger, that the association of total cholesterol with RNFL thickness became less strong, and that the association of antihypertensive medication with RNFL thickness became less strong. Then, when we replaced educational level with occupational level or income level, we found less strong associations of cardiorespiratory fitness with RNFL thickness. Sixteenth, for the three associations under study that were modified by glucose metabolism status, when we tested whether interaction terms composed of continuous measures of glycaemia also modified these associations, we found that two associations were modified by all three continuous measures and one associations was modified by two out of three continuous measures (Supplementary Table 12). Last, after full adjustment (model 3), greater age was significantly associated with lower retinal sensitivity and lower RNFL thickness (standardized beta [95% confidence interval], per year greater age -0.04 [-0.05; -0.04] and -0.01 [-0.01; -0.01], respectively; Supplementary Table 8). Hence, the beta for 1 SD greater HbA1c corresponds with approximately 1.3 year of aging for retinal sensitivity and 5.0 years of aging for RNFL thickness; the beta of 1 SD lower adherence to a healthy diet corresponds with approximately 1.5 year of aging for retinal sensitivity and 3.0 years of aging for RNFL thickness; and the beta of 1 SD lower cardiorespiratory fitness corresponds with approximately 1.3 years of aging for retinal sensitivity and 3.0 years of aging for RNFL thickness. Therefore, added up, the combination of these three

adverse factors corresponds with approximately 4.1 and 11.0 years of “aging” for, respectively, retinal sensitivity and RNFL thickness.

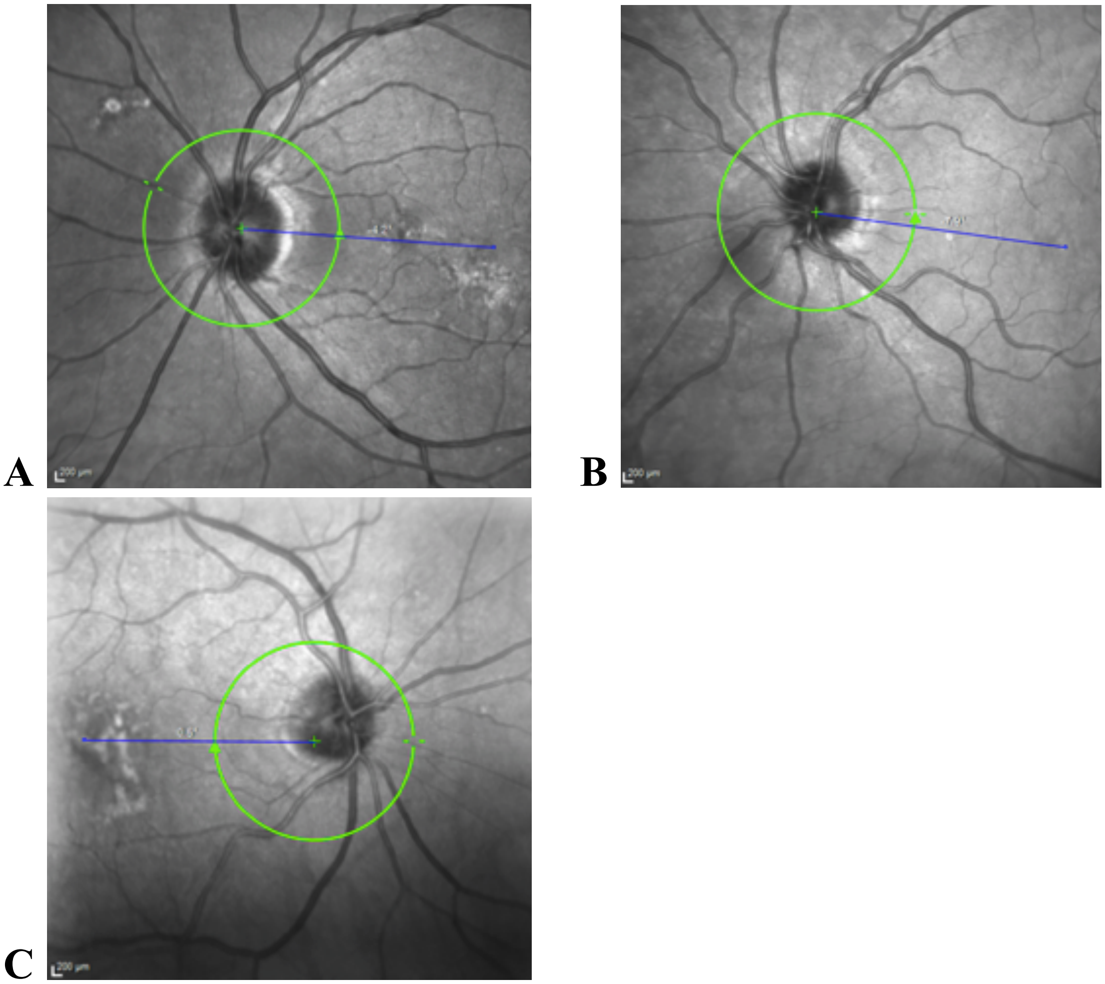

Supplementary Figure 1. Examples of quality of centering of circular scans on the optic nerve head. A) good quality, B) poor quality, C) very poor quality.

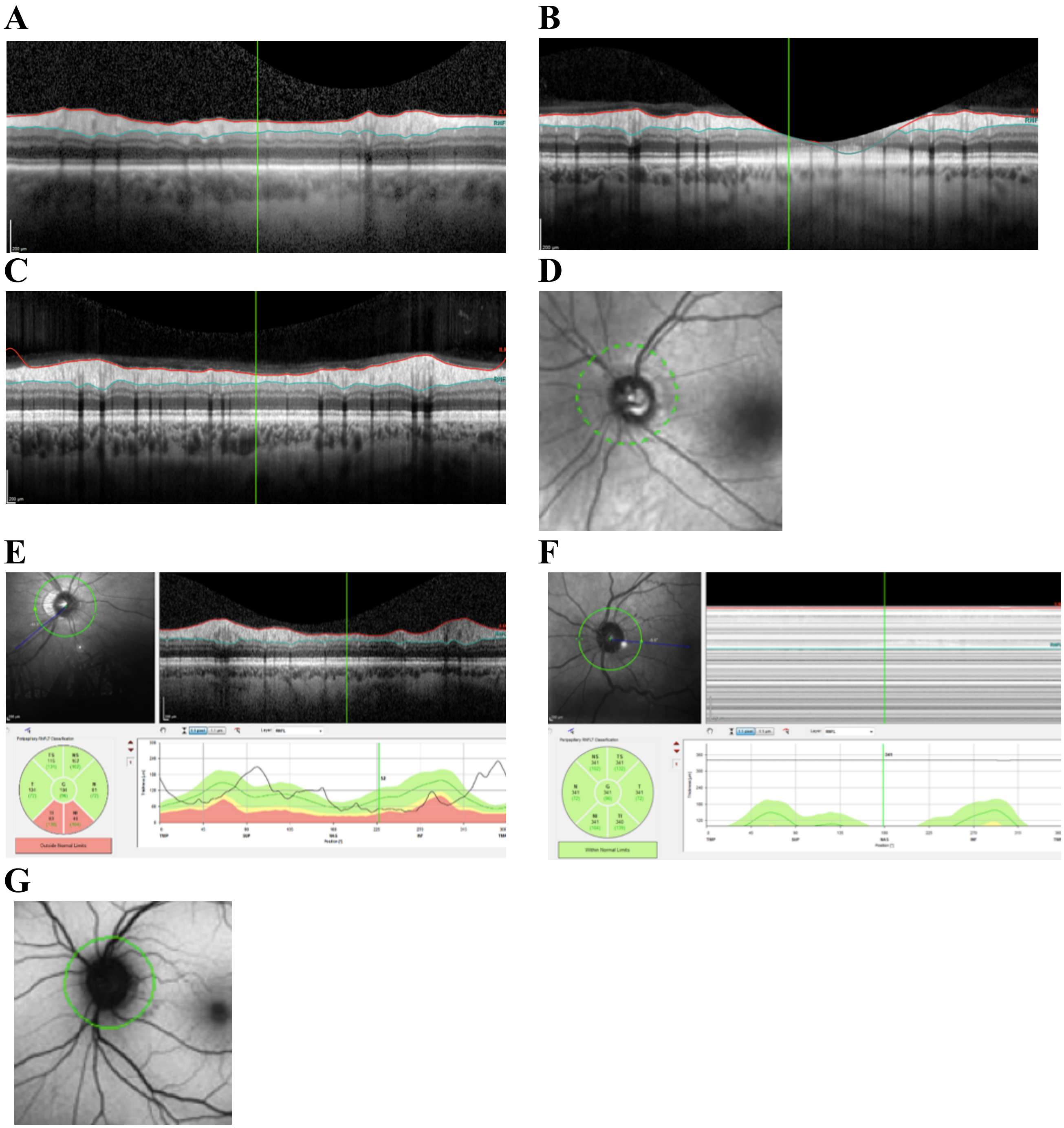

Supplementary Figure 2. Examples of poor quality and scan errors. A) Example of poor imaging quality (Signal-to-noise ratio<15 dB); B) OCT device too close to the eye; C) RNFL layer incorrectly defined; D) incorrect circle position (dashed line); E) participant does not look in the correct direction; F) technical problem with OCT device; G) autofluorescence on. OCT, optical coherence tomography; RNFL, retinal nerve fiber layer thickness.

A

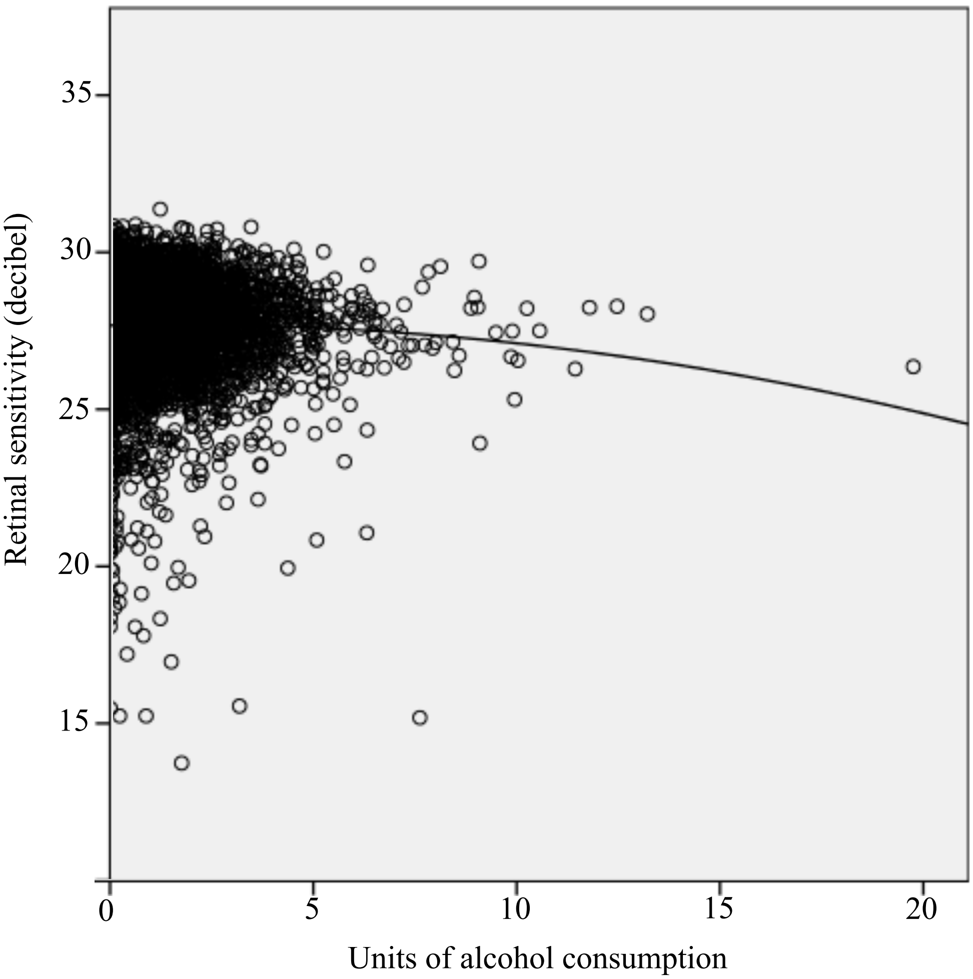
Retinal sensitivity (decibel)
35
30
25
20
15
0
5
10
15
20
Units of alcohol consumption

B

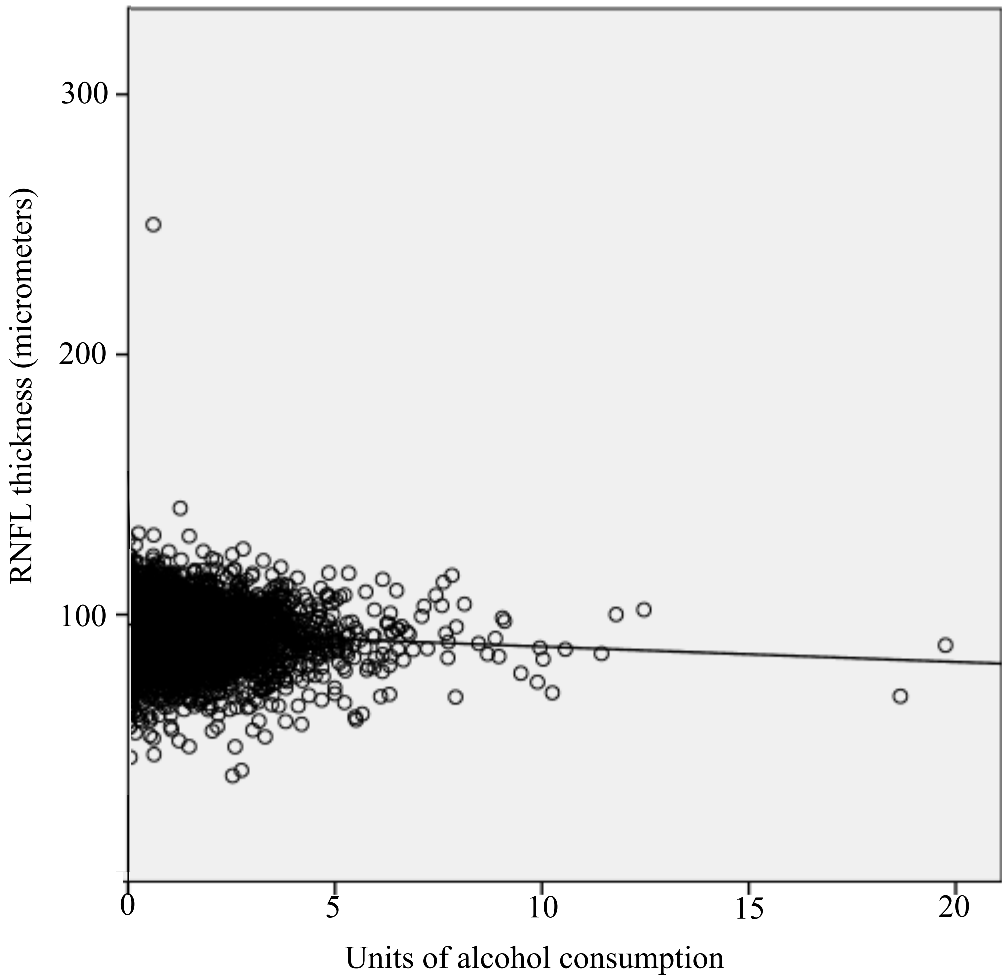

Supplementary Figure 3. Panel A shows the quadratic (i.e., non-linear) association of alcohol consumption with retinal sensitivity and panel B shows the linear association of alcohol consumption with RNFL thickness. For both figures, one unit of alcohol consumption corresponds with 10 grams of ethanol intake/day. RNFL, retinal nerve fiber layer.
